# Preferences for home care to enable home death among adult patients with cancer in late palliative phase – a grounded theory study

**DOI:** 10.1186/s12904-022-00939-y

**Published:** 2022-04-11

**Authors:** Toril Merete Nysæter, Cecilia Olsson, Tuva Sandsdalen, Bodil Wilde-Larsson, Reidun Hov, Maria Larsson

**Affiliations:** 1 Department of Health and Nursing Sciences, Inland Norway University of Applied Sciences, 2400 Elverum, Norway; 2 Department of Health Sciences, Karlstad University SE, Karlstad, Sweden; 3 Department of Bachelor Education, Lovisenberg Diaconal University College, Oslo, Norway; 4 Centre for Development of Institutional and Home Care Services (USHT), Inland (Hedmark), Elverum, Norway

**Keywords:** Patients' preferences, Cancer, Palliative care, End-of-Life (EOL) Care, Home death, Grounded Theory

## Abstract

**Background:**

The wish to be cared for and to die at home is common among people with end-stage cancer in the western world. However, home deaths are declining in many countries. The aim of this study was to explore the preferences for home care over time to enable home death among adult patients with cancer in the late palliative phase.

**Methods:**

A qualitative method was applied according to grounded theory (Corbin & Strauss, 2008). Data was collected using individual interviews (*n* = 15) with nine adult patients. One to two follow up interviews were conducted with four patients. Sampling, data collection and constant comparative analysis were undertaken simultaneously.

**Results:**

The findings are presented as a conceptual model of patients’ preferences for care to enable home death. The core category “Hope and trust to get the care I need to die at home” showed that the preference to die at home seemed stable over time and did not change with deterioration in health status and progression in illness. Five categories were related to the core category. The categories “being in the present”, “be safe and in charge” and “be seen and acknowledged” describe the patients’ preferences to live a meaningful life until death and be the same person as always. These preferences depended on the categories describing characteristics of healthcare personnel and the organisation of care: “reliable, compassionate and competent healthcare personnel” and “timely, predictive, continuous and adaptive organisation”.

**Conclusion:**

An important preference over time was to be here and now and to live as meaningful a life as possible until death. Moreover, the patients preferred to retain control over their lives, to be autonomous and to be seen as the person they had always been. To achieve this, person-centred care provided by healthcare personnel with competence, skills and enough/ample time were required. In addition, home care needed to be organised in a way that ensured continuity and predictability. Systematic implementation of a person-centred care model and the use of advanced home care plans with continued re-evaluation for patients’ preferences of home care were proposed measures to enable home death.

## Background

Most people in the western world, including terminally ill patients with cancer, express a wish to receive End-of-Life (EOL) care at home and to die at home [1–3]. Home death has therefore been proposed as a measure of quality in EOL care [4–6]. Nonetheless, the number of home deaths is declining in most countries [7, 8]. However, in a few countries the number of home deaths has increased in recent decades. In Canada and the UK, almost every third person dies at home, and in the Netherlands every second [9–11]. In these countries, including the United States (US), policy changes and an increased focus on home-based models of hospice and palliative care have contributed to the development [1, 11]. In other countries, e.g. Norway [11], regardless of political initiatives for increased opportunities for people to be cared for and die at home, the number of registered cases has declined.

The vast majority of studies investigating the preferred place of death among patients with cancer are conducted with a cross sectional design in the early palliative phase. It is not clear whether the preference to stay at home remains constant over time and whether the patients receive the preferred care to enable home death [1, 3]. Moreover, these studies were performed with the patient’s family and relatives, mostly bereaved family caregivers, as proxies. Using family caregivers as proxies can give insight into the final terminal phase when patients are unable to communicate their preferences [12, 13]. These studies show that there is room for improvements regarding symptom management, information and self-determination [14, 15]. However, whether there is congruence between the patients’ and relatives’ perceptions is unclear. Studies using healthcare personnel as proxies show that nurses tend to overestimate the importance of trust and empathy and to underestimate the importance of nursing skills, provision of equipment and access to care [16].

Even if one might argue that patients in the late palliative phase are a vulnerable group, it is necessary to hear the patients’ voice in order to enable care that meets their needs and preferences. Accordingly, a review by Sandsdalen et al. [17] concludes that it is important that during their last year of life, patients can live a meaningful life as autonomously as possible. Moreover, they prefer person-centered care provided with compassion and respect and characterised by continuity and timely support. However, preferences for home care to enable patients with cancer in the late palliative phase to die at home, were not the focus of this review. In addition, some studies reveal uncertainties to whether the wish to die at home is constant over time when death is approaching [1, 3, 18–21] and that influencing factors appear to be illness progression, healthcare resources and repeated communication during the trajectory of care regarding wishes as to place of death [22]. As there is increased focus on patients’ perspectives in developing, improving and measuring the quality of health care services, not least in palliative care settings, it is important to explore patients’ preferences regarding care during all phases of palliative care, including near the end of life [4, 16, 17]. Therefore, in this study we aimed to explore the preferences for home care over time among adult patients with cancer in the late palliative phase to enable home death.

## Method

### Design

This study has an exploratory design, as there is limited knowledge concerning preferences for home care among patients with cancer in the late palliative phase to enable home death. To gain a better understanding concerning of the patients’ perspective concerning the phenomena and to construct a conceptual model we used grounded theory (GT) [23]. The Corbin and Strauss (2008) method was deemed appropriate for the explorative aim of this study as it is a qualitative research method that draws out concept, models or theories from empirical data. The study is reported in accordance with Consolidated criteria for reporting qualitative research (COREQ) recommendations [24].

### Setting

The study was conducted in six different municipalities in two counties in Norway. A municipality is a council area that can be either a town/city or a district with its own government. The patients were recruited from both urban and rural municipalities in the southeast of Norway [25]. The Norwegian municipalities [26] are responsible for providing health care services including palliative home care services to meet the needs of each patient [27]. Palliative care is organised as specialised and non-specialised care, whereby the latter mainly serves patients in the municipalities, as an integral aspect part of community care [28]. Community care is responsible for and serves patients both in nursing homes and at home, via home care and their general practitioner [28]. In addition, the municipalities in this study have cancer nurses employed as cancer care coordinators, which are informed about all patients with a cancer diagnosis within their district area.

A cancer care coordinator is responsible for coordinating care, and providing continuity and support to patients and relatives throughout the trajectory of care [29].

### Participants and recruitment

Written permission was obtained from the head of the home care services before the study was conducted. The cancer care coordinators were then informed about the study and asked to find potential patients in order to carry out the data collection.

The patients that were included met the criteria of being adult (at least 18 years of age), with cancer in the late palliative phase (expected survival 6—12 weeks, using The Surprise Question [30]), were informed and aware of their state of illness and prognosis, had no cognitive impairment, could understand and speak Norwegian, were living in their own home alone or with relative(s) had an expressed wish to die at home documented in the patient record. The patients were to receive care and support from cancer care coordinators, and they could receive home care. Patients living in nursing homes were excluded.

The cancer care coordinators identified eligible patients and informed them about the study both verbally and in writing. If the patient expressed interest in the study, they were given time (24–48 h) to think to consider whether to participate. All patients that were informed about the study accepted participation (*n* = 11). After inclusion, the health condition of two patients deteriorated rapidly and an interview were not possible before they died. When a patient gave their consent to participate, the first author was informed. The first author (TN) then contacted the patients via telephone and further informed them about the study and made appointments for the time and place of the first interview. All patients provided informed written consent. The interviews were conducted in a private place in the patient’s home, except for one interview that, for practical reasons, was held in a hospital care centre. Nine patients, two women and seven men, participated in the study. The patients age ranged between 47 and 90 years (median 71 years). All patients were ethnic Norwegian (Table 1). At the time of recruitment, all patients received care and support from the cancer care coordinator on a weekly or monthly basis and six patients received home care one to six times daily. Experience of home care prior to inclusion in the study varied among the patients. Five had received home care between one month and six months while one had been receiving home care for several years due to needs related to a chronic disease.

**Table 1:** Characteristics of participants (*n* = 9)

Characteristics	Number
**Sex**	
Women	2
Men	7
**Age (years)**	
Median	71
Range	47—90
**Marital status**	
Married / cohabitant	6
Living alone	3
**Education level**	
Elementary School	4
Secondary school	2
University college / university	3
**Living conditions**	
Detached house	5
Semi- detached/multi-storey	4
**Ethnicity**	
Ethnic Norwegian	9

### Data collection

Data collection was performed as individual interviews between April 2018 and March 2019. All interviews were conducted and recorded by the first author (TN). TN is a specialist nurse in cancer care, educated and trained in Clinical Supervision and communication and a PhD student.

An interview guide was developed by the authors with topics concerning the patient’s preferences for care, how they experienced the care given, whether they got what they preferred and how it was consistent with their wishes for future care and support [23]. The interviews started as an open dialogue in which the patient was encouraged to talk freely about their experiences and preferences regarding their home care. The interviews were consequently guided with open questions such as who, which, what etc. in order to narrow or extend the field of interest. To maintain an open dialogue during the interview, the interview guide was used as a reminder, to make sure the topics were covered.

The interview guide was changed and modified during the data collection and data analysis process, focusing on the emerging categories and their properties and dimensions [23]. At the beginning of the recruitment the sampling was purposeful, as the recruiting nurses asked patients that met the inclusion criteria. As the inclusion process proceeded, theoretical sampling was used in order to saturate the emerging categories during the data analysis [23].

The nine patients were interviewed between one to three times. In total 15 interviews were conducted. The follow-up interviews were conducted when there was a deterioration in the patient’s illness status in order to explore whether this led to changes regarding preferences for care and support and the wish to die at home. For example, a follow-up interview could be conducted in connection with the onset of distressing symptoms such as pain that needed professional management, general infections or patients' self reported experiences of that death was approaching. The patient or the family caregiver contacted the first author when a change occurred, and a time for a follow-up interview was agreed. The time between the initial interview and follow-up interviews were one to three weeks.

The first nine interviews lasted from 23 min up to one hour and 45 min (median 47 min). Four patients were interviewed twice. These were both men and women cohabitating with someone (*n* = 3) and living alone (*n* = 1). From among these, two patients, one man cohabitating and one woman living alone, were interviewed a third time. The follow-up interviews lasted between 20 min and one hour and 39 min (median 59 min). In connection with the interviews, field notes were written about the interviewer’s immediate impressions and thoughts during and directly after the interviews.

### Data analysis

Data collection and data analysis were performed in line with the constant comparative method in grounded theory [23, 31]. The interviews were audio-recorded and transcribed verbatim as soon as possible after each interview by the first author (TN). The field notes were included in memos. The open coding started after reading through the entire interview, and memos. Open coding was carried out by hand to ensure closeness to the data material. The text, interviews and memos, was investigated line by line to identify meaning and process. In the first step, codes and concepts were identified, named and constantly compared, contrasted and grouped into preliminary categories (TN, BWL, ML).

All authors contributed in the next steps of the analysis (TN, CO, TS, BWL, RH and ML). The emerging categories were named and further developed by asking the questions: “what? who? where? how? and when?” to the text. In addition, temporal and spatial questions were asked about the text and the properties and dimensions in the emerging categories [23]. In the axial coding procedure connections between categories were sought for and the categories were further clarified. In the selective coding process, each category was saturated with information from new interviews or interviews already analysed [31]. Memos were written during the entire process to ensure that thoughts, reflections and preliminary connections that were discussed in the research team were included. Finally, a core category was identified, and a conceptual model was established.

## Results

**Fig. 1:**
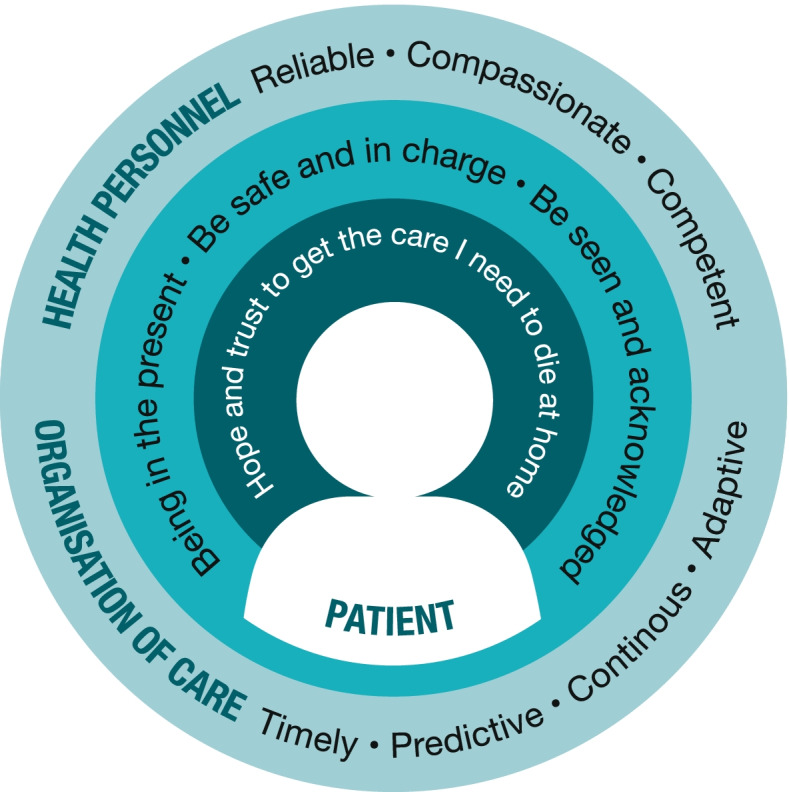
Conceptual model of the preferences for home care over time among adult patients with cancer in the late palliative phase to enable home death

The findings are captured in the core category “Hope and trust to get the care I need to die at home”. The core category showed that the preference to die at home was stable over time and did not change with deterioration in health status and progression in illness. The categories “Being in the present”, “Be safe and in charge” and “Be seen and acknowledged” describe patients’ preferences to live a meaningful life until death, to be seen and valued as the same person as always, and to be in charge and controlling their own lives. These preferences were dependent on the categories “Reliable, compassionate and competent healthcare personnel” and “Timely, predictive, continuous and adaptive organisation”, which described patients’ preferences related to characteristics of the healthcare personnel and organisation of care. The core category together with the five categories built up a conceptual model of preferences for home care to enable home death among adult patients in the late palliative phase (Fig. 1).

### Hope and trust to get the care I need to die at home

From an overall perspective the patients expressed that they had hope and trust in the healthcare personnel and the health care system, to provide care enabling fulfilment of their wish to die at home. This also included patients that had earlier negative care experiences. When the illness progressed over time, and the need for care and support changed in line with negatively affected bodily functions, the wish to die at home remained. Hence, the patients repeatedly interviewed, continued to express a wish to get EOL home care and to die at home. Moreover, the hope and trust to get the care they preferred in accordance with their needs were maintained. The patients stated that a factor contributing to their trust in their preferences for care being met, was their explicitly expressed wish to die at home. The fact that the patients knew that the healthcare personnel were aware of the patient´s individual wish to die at home strengthened patients’ confidence in receiving care adapted to their preferences and needs until death.

### Being in the present

Being in the present concerns being here and now, taking one day at a time and living each day until death. The patients expressed that being in the present was a prominent and stable preference, that did not change over time. Being in the present was thus equally important, despite the progression of illness and deterioration in health status.

The patients were informed about, and had accepted, that death was imminent. Therefore, it was important to live each day as full and meaningful as possible.

*“It’s important for me to take one day at a time. It’s important that I don’t stress to have a good day – but try to fill the day with something meaningful. I’m not afraid of death, and I don’t spend much time to think about it. What happens, is happening to my body and I’ve accepted it.” Patient 5, age 47, interview 1.*

In accordance with being in the present and living each day, they did not want to talk about death on a daily basis, because it reminded them that life was to end and this, in turn, affected their ability to find life here and now meaningful to live.

### Be safe and in charge

The patients described that they preferred to stay and be cared for in their own home as this created a sense of security and was an arena in which they felt in charge of their own lives. The patients expressed how they preferred to take care of themselves as far as they could, as this strengthened the sense of being in charge of their own lives and contributed to a feeling of normality.

*“I take care of myself in the morning even though they come from the home care services to help me. They`re here to help me, but I think that it`s really ok to take care of myself as far as I can. But it feels safe to know that they will visit me.” Patient 2, age 82, interview 2.*

As the illness progressed and the ability to take care of themselves decreased it became more and more important to do as much as they could manage themselves.

The patients emphasised the importance of healthcare personnel being aware of their expressed wish to die at home. They felt certain that the healthcare personnel would provide care and facilitate their homes with medical equipment, to fulfil their wish to stay at home as death became imminent.

*“The occupational therapist said that even though we were living in a detached house with two floors it would still be possible to have everything on one floor. I can have a wheelchair ramp or something… to adapt the house so I can stay here longer.” Patient 5, age 47, interview 1.*

Patients who received home care and were living alone expressed how changes related to the progression of the illness over time, such as acute situations or changes related to self-care abilities, threatened their feeling of being safe and in charge.

### Be seen and acknowledged

The patients preferred healthcare personnel who saw and acknowledged them as persons by being “here and now”, and by listening and having conversations and enough time to do what was needed. Since no two days were the same and their needs varied every day, small events such as helping to hang up laundry became of great importance. This was more important for those who lived alone, were unable to participate in activities and had a limited family and / or social network, so that healthcare personnel were an important part of their day.

“*I really notice that the people who work here are interested in me and they truly wish me all the best. And it feels so good.” Patient 8, age 85, interview 1.*

When progression of the cancer disease required changes in the home situation, such as facilitating their home with various medical equipment or personalised pain management, the need for dialogue about approaching death became evident. The patients preferred to these dialogues with healthcare personnel who, they felt saw them as an autonomous person and involved them in decisions about their care and treatment.

### Reliable, compassionate and competent healthcare personnel

The patients described reliable, compassionate and competent as important characteristics of healthcare personnel. Healthcare personnel with these characteristics contributed to enabling a sense of safety and security in everyday life. The patients preferred healthcare personnel who came at the agreed time and kept their promises. The patients also preferred healthcare personnel who showed compassion by being there for them in situations they experienced as fearful or threatening, such as medical procedures they experienced as unpleasant. The healthcare personnel's competence was described as twofold. On the one hand, level of traning was highly valued when it came to technical procedures and assessment of the patients' illness, symptoms and needs. When changes in the illness occurred, for example extended pain or general infections, they relied on healthcare personnel to be able to see these changes and perform actions by reporting to the personnel for the respective area of responsibility, for example GP or the palliative care team. They expressed it as a burdensome if they had to report changes themselves. Cancer care coordinators were considered to be highly competent, based on the role they held, even though the patients only met them weekly or monthly.

*“It feels very safe and secure. If there are any questions, I can ask her and then I will get answers and help as far I can.” Patient 9, age 90, interview 3.*

On the other hand, competence was related to being competent in the sense of being familiar with the patient´s situation and their home. They preferred healthcare personnel with whom they had a good and well-established carer-patient relationship and who were familiar with their home.

Some of the patients, of both genders, expressed how they preferred and were more comfortable with older, mature female healthcare personnel who they considered to be more reliable, competent and compassionate than their younger colleagues.

*“These elderly, mature ladies who are here, I must say that they are a bit more compassionate. They ask me ´how are you feeling now? ´ and ´how are you?´ and so on. This makes me feel extra safe and secure.” Patient 2, age 82, interview 2.*

### Timely, predictive, continuous and adaptive organisation

The organisation of care had an impact on patients’ everyday lives. The patients preferred the healthcare personnel to come at the agreed time, as this ensured predictability and security in their everyday lives. They also preferred continuity in the staff group and knowing which healthcare personnel would come on the next occasion. Patients who received home care up to several times a day experienced meeting many different people and healthcare personnel from different professions as demanding.

*“Sometimes I wished that they weren’t so many so I could have a closer relationship with them. Because you lie here thinking, who is coming now, who is coming to give me my injection? It`s so uncertain. Because there is someone special that I wish would come back. I often ask them: will you come back today? Because I want that special one to return.” Patient 8, age 85, interview 1.*

The patients described this unpredictability as stressful and they experienced feelings of drained energy, anxiety and irritation.

Due to changes in the illness, the patient’s situation and need for care could alter rapidly. The patients preferred a health care service organised in a way that enabled immediate adaptation of the care when a change occurred. If the patients experienced a lack of adaptability, they described how they felt disappointed and insecure. Predictive, reactive and rapidly adaptive care seemed to be particularly important for patients living alone.

## Discussion

This study explored preferences for EOL home care over time among adult patients with cancer in the late palliative phase to enable home death. The main finding is that despite deterioration in their illness and approaching death, patients continued to put their hope and trust in getting the EOL care that would enable them to stay at home and finally, to die at home. Accordingly, the findings indicate that patient’s preference to die at home remained stable over time. However, the sample in this study is small and there is a need for more research to confirm this. The patients emphasised that they preferred EOL home care that allowed them to live each day until death and supported their ability to manage fundamental care needs as far as possible. The patients stressed the importance of being seen as a person and being in charge and having control of their own lives until death. Crucial prerequisites were reliable, compassionate and competent healthcare personnel and the care being timely, predictive and continuous.

Studies show that home is the preferred place to die among patients in the early palliative phase [1–3], therefore home death has been proposed as a measure of quality in EOL care [4–6]. Yet, despite this, and policies advocating home care at EOL, few people die at home, particularly in Norway. Home has been shown to represent familiarity, with potential for normalcy and a safe haven when death is approaching [32]. The patients in the current study expressed how being at home facilitated participation in decisions regarding their own care and promoted feelings of autonomy. Furthermore, being at home enabled them to be in the present and to live a meaningful life until death, which was stressed as important by the patients. Knowing that death was imminent made it even more important to be in the present. It is well-known that to be able to go on with one’s life is even more important in the last stages of life [17, 33]. The current study contributes more evidence in support of this knowledge.

Socioeconomic factors, age and gender have in earlier studies been found to affect patients’ preferences for place to die [34, 35]. Accordingly, being older, living alone and being a woman has been shown to be negatively associated with home death [36, 37]. In our study, an inclusion criterion was an expressed wish to die at home, and no patient changed their mind. However, inclusion of more women and patients living alone might have given another result. In addition to the skewed gender distribution of the patients, there is a large range in age (47–90 years) of the patients participating in our study. Earlier studies have shown that old age is negatively associated with home death in cancer [36, 37], but we did not find any such tendencies in our study.

Earlier negative experiences of home care have been shown to be associated with non-fulfilment of patients’ preference for home death [36]. Furthermore, in our study some patients described negative home care experiences. However, they still retained the hope and trust that they would get the care they needed to make it possible to die in their own home. One explanation could be that they had explicitly expressed their wish to die at home repeatedly to the healthcare personnel in combination with relational continuity in care. Studies have shown that when conversations about patients’ preference to die at home are held, it is more likely that home death will be achieved [36, 38]. Unfortunately, healthcare professionals are often reluctant to enter into conversations with patients concerning place of death [39, 40]. Consequently, efforts must be made to ensure that healthcare personnel working in EOL care have good communication skills. It is crucial that they have the ability to create and engage in a dialogue in which patients themselves can express what contributes to meaning, dignity and relief from distress, and acceptance of their values and beliefs. Furthermore, in our study the patients knew that death was imminent. Honest information and dialogue about health status and progression of illness is a prerequisite for making adequate choices for the future [41]. Conversations in line with these concepts may contribute to insight into and fulfilment of the patients’ preferences [42].

However, the patients in our study did not want to have daily talks about death, as they believed this served as a constant reminder that their life was shortened and fostered thoughts about how that life was meaningless to live. Therefore, our study highlights the importance of establishing an advanced home care plan and of having timely conversations when changes occur, in addition to the initial conversation about death, including preferred place of death. Consequently, future intervention studies need to take this knowledge in to account.

Previous studies have described the importance of availability and relational continuity in palliative home care [29, 43, 44], which was also confirmed in our study, as the patients stressed that they wanted to meet the same healthcare personnel. The establishment of a solid patient-carer relationship proved to be crucial for the patients in this study, to hope and trust they would get the preferred care and achieve a home death. The patients relied on the cancer care coordinators’ competences in coordinating care and assessing the patient’s needs and symptoms. Freijser et al. highlight the cancer care coordinator’s role as a primary point of contact providing relational continuity [45]. A European study with participants from five countries has also pointed out that a small team of trusted healthcare personnel who are available, provide multidisciplinary care and regularly transfer information to all personnel involved was a prerequisite for continuity in care [43]. However, the patients in our study expressed a lack of relational continuity with the healthcare personnel, especially during inconvenient working hours when nurse assistants were mostly responsible for the direct care. These results are in line with earlier studies [42, 46, 47] and highlight that improvements in the organisation of and access to the home care services are needed. In addition, Oosterveld et. al emphasise the need for improvements regarding professional collaboration and information transfer between professionals for high quality home care [48]. A meta-ethnographical study emphasises that palliative home care teams who are available and empowered with the resources to provide competent care increases the patient´s sense of security [49]. Moreover, availability and relational continuity are prerequisites for person-centred palliative care [42]. To be seen and acknowledged as a person was of utmost importance for the patients in this study to feel safe and secure, thereby enabling home death. One model that can be used to achieve person centered palliative care is the 6S model [42]. This care model is based on the notion that care is co-created between the patient and the healthcare personnel, which enables the patient to live as meaningful and dignified a life as possible until death. The intention with the 6S model is to integrate the physical, psychological, social and existential needs of patients, and to be a tool for the nursing process, i.e. planning, documentation, interventions, evaluation, and transfer of information between caregivers. Future interventions studies that evaluate the effect of the 6S model meeting patients’ preferences for palliative home care that enables home death would be of interest for patients, family caregivers and healthcare personnel, as well as for policy makers.

### Methodological considerations

Corbin and Strauss [23] have proposed criteria for evaluating the quality of a GT – study and we sought to follow these as sincerely as possible. These criteria concern the research process and empirical grounding of the study, with clear descriptions of sampling procedures, theoretical sampling, concept generation, emerging categories and variation in the theory. In the description of the method, we have sought to give a clear explanation of how these steps were carried out.

There are some limitations in the study. The sample size (*n* = 9), can be questioned as it might be considered small. However, given that the patients represent a vulnerable group, the number of patients finally included could be considered to be appropriate. A contributing factor might be gatekeeping. Gatekeeping, which is difficult to both predict and handle, is a well-known phenomenon, where the emphasis is to protect those who are vulnerable and consequently patients are not asked about participation, withholding them the opportunity to decide for themselves [50]. Furthermore, the “temporal over time” aspect can be questioned as only four out of nine patients were interviewed more than once. Follow-up interviews with the other patients was not possible to conduct due to rapid deterioration in illness. However, the number of patients finally included, and follow-up interviews conducted, could be considered to be appropriate as categories were densified and the axial- and selective coding processes could be carried out successfully.

Another limitation in the study was the skewed gender distribution of the patients. However, during the recruitment process, there were fewer women meeting the inclusion criteria, which is in line with previous studies showing that fewer women than men die at home [7, 51]. Despite a skewed sample due to gender, both women and men, living alone or cohabitating, are represented in the follow up interviews and this strengthens the results.

The sample in our study, mirrors patients with cancer in the late palliative phase who have expressed a wish to die at home. Initially, we used purposeful sampling, where the sampling and analysis persisted as theoretical sampling followed. This entailed that an additional number of patients were included who could add variation to the experiences, in order to saturate the categories with various dimensions.

When data from the interviews did not contribute to new categories, this was interpreted as a sign of category saturation. Since it is difficult to estimate the issue of saturation in categories, this was critically discussed, and the research group agreed that this point had been reached. In addition, before and during the data analysis the research group discussed their own preconceptions about the subject in focus and used critical reflection throughout the research process in order to find the hidden meanings.

## Conclusion

The findings of this study indicate that the preference to die at home remain over time among adult patients with cancer in the late palliative phase regardless of deteriorating health status and aggravated symptoms. However, considering the limited number of patients on which this finding is based, more studies are needed before definitive conclusions can be drawn. We stress the importance of repeated dialogues regarding preferences for EOL home care and preferred place of death throughout the care trajectory also during the late palliative phase.

Despite their deterioration and increased care needs, the patients in this study continued to trust that the healthcare personnel would provide EOL home care enabling them to stay at home until death. The patients preferred EOL home care that made it possible to be in the present, to be here and now and live as meaningful a life as possible until death. Moreover, the patients stressed that they preferred EOL home care that allowed them to maintain control over their own lives, be autonomous and be seen as the person they had always been. To achieve this EOL home care, person-centred palliative care provided by healthcare personnel with adequate competence and skills are needed, together with a care organisation that enables continuity and predictability of care, as well as both timely care and ample time. Therefore, the implementation of person-centred care and use of advanced home care plans with continued re-evaluation of patients’ preferences for EOL home care are measures proposed to enable home death. As home deaths cannot be achieved without informal family carers there is also a need for studies concerning the family caregivers' preferences for EOL home care.

## Data Availability

The dataset generated and analysed for this study are not publicly available because the data are confidential due to the participants´ consent did not include sharing the dataset and the conditions of NSD (Norwegian Centre for Research Data). Request for access to data is made to the corresponding author.

## Abbreviations

EOL: End-of-Life care
